# Different chromatin and DNA sequence characteristics define glucocorticoid receptor binding sites that are blocked or not blocked by coregulator Hic-5

**DOI:** 10.1371/journal.pone.0196965

**Published:** 2018-05-08

**Authors:** Brian H. Lee, Michael R. Stallcup

**Affiliations:** Department of Biochemistry and Molecular Medicine, Norris Comprehensive Cancer Center, University of Southern California, Los Angeles, California, United States of America; Università degli Studi di Milano, ITALY

## Abstract

The glucocorticoid receptor (GR) regulates genes in many physiological pathways by binding to enhancer and silencer elements of target genes, where it recruits coregulator proteins that remodel chromatin and regulate the assembly of transcription complexes. The coregulator Hydrogen peroxide-inducible clone 5 (Hic-5) is necessary for glucocorticoid (GC) regulation of one group of GR target genes, is irrelevant for a second group, and inhibits GR binding to a third gene set, thereby blocking their regulation by GC. Gene-specific characteristics that distinguish GR binding regions (GBR) at Hic-5 blocked genes from GBR at other GC-regulated genes are unknown. Here we show genome-wide that blocked GBR generally require CHD9 and BRM for GR occupancy in contrast to GBR that are not blocked by Hic-5. Hic-5 blocked GBR are enriched near Hic-5 blocked GR target genes but not near GR target genes that are not blocked by Hic-5. Furthermore blocked GBR are in a closed conformation prior to Hic-5 depletion, and require Hic-5 depletion and glucocorticoid treatment to create an open conformation necessary for GR occupancy. A transcription factor binding motif characteristic of the ETS family was enriched near blocked GBR and blocked genes but not near non-blocked GBR or non-blocked GR target genes. Thus, we identify specific differences in chromatin conformation, chromatin remodeler requirements, and local DNA sequence motifs that contribute to gene-specific actions of transcription factors and coregulators. These findings shed light on mechanisms that contribute to binding site selection by transcription factors, which vary in a cell type-specific manner.

## Introduction

Transcriptional regulation is a major factor regulating cellular activities and thereby in determining human health and disease. Transcriptional regulation is initiated by transcription factors which bind to specific DNA enhancer and silencer elements that control transcription of the associated genes. Transcription factors recruit coregulator proteins, which carry out chromatin remodeling and regulate the formation of an active transcription complex at the transcription start site (TSS). Many coregulators cooperate to accomplish these tasks, which lead to increased or decreased mRNA synthesis [1–3]. It is particularly noteworthy that different genes regulated by the same transcription factor in a particular cell or tissue type require different combinations of coregulators. Thus coregulators function in a gene-specific manner, and each coregulator is required for the regulation of a subset of the genes regulated by any given transcription factor [2, 4–6]. However, the site-specific characteristics at the transcription factor binding sites that determine the recruitment of and/or requirement for specific coregulators are mostly unknown. Here we report site-specific characteristics of chromatin and DNA sequence that dictate gene-specific effects by a coregulator named Hydrogen peroxide-inducible clone-5 (Hic-5, also known as *TGFB1I1*) as it cooperates with the glucocorticoid receptor (GR, *NR3C1*). GR is a nuclear receptor family transcription factor, activated to regulate gene transcription by the natural glucocorticoid (GC) hormone cortisol and a variety of synthetic cortisol analogues, which are frequently used to treat inflammatory diseases and some types of cancer [7]. Among many physiological pathways regulated by GR and GC are inflammation and metabolism of glucose, lipids, and proteins, which they control by activating and repressing transcription of specific genes.

Hic-5, which belongs to the paxillin protein family, functions in the cytosol as an adapter protein at focal adhesion complexes [8]. However, Hic-5 is also found in the nucleus where it is a coregulator for several transcription factors including GR [9–15]. In its role with various nuclear receptors, Hic-5 has been associated with epithelial cell differentiation [10], endometriosis [11], and prostate tumorigenesis [12]. As is typical for coregulators, the actions of endogenous Hic-5 are highly gene-specific; Hic-5 helps GR to activate some target genes and repress others. GC-regulated genes can be divided into three different classes based on the actions Hic-5: one class of genes (*ind*) are regulated by GC independently of Hic-5; the Hic-5 modulated (*mod*) genes are regulated by GC when Hic-5 is present in cells, but their regulation is significantly altered or sometimes eliminated when Hic-5 is depleted; finally, the expression of a third gene class (*block*) is blocked by Hic-5, but they become GC-regulated once Hic-5 is depleted [6, 16]. In a previous study we analyzed a few genes in each class and found that Hic-5 is recruited to GR binding regions (GBR) of GC-induced *mod* genes by its direct interaction with GR; for those genes Hic-5 promotes transcription complex assembly by facilitating recruitment of the Mediator complex and RNA polymerase II [6]. However, for the three selected *block* genes, Hic-5 prevented GC-induced chromatin remodeling and robust GR binding to GBR close to the *block* genes [6, 16].

Chromatin structure plays a major role in GR occupancy. GR and other transcription factors interact with the DNA binding site in a dynamic on and off manner [17, 18]. GC-induced binding of GR to GBR is a cooperative process between GR and chromatin remodelers, in which GR at first recognizes its specific DNA binding motif weakly (due to restrictive chromatin conformation) and recruits ATP-dependent chromatin remodeling enzymes that open up chromatin structure allowing more robust GR occupancy [16, 19–21]. The discovery that Hic-5 prevents transcription of *block* genes by impeding GR occupancy and GC-regulated chromatin remodeling at those genes, but not at *mod* or *ind* genes, suggests that different chromatin remodelers may be required for robust GR binding at GBR associated with *block* genes versus *mod* and *ind* genes. Therefore, in the current study we assessed global GR binding sites to determine the gene-specific characteristics that specify the actions of Hic-5 to prevent GR from binding to and regulating transcription of *block* genes, while in the same cells there is robust GR binding to and GC-regulated transcription of the *ind* and *mod* classes of GR target genes. We tested the hypothesis that gene-specific actions of coregulators are determined by the chromatin environment and DNA sequence motifs specifying potential binding sites for other transcription factors around the GBR, and that Hic-5 influences GR occupancy by interfering with the dynamic interaction of GR with specific chromatin remodeling complexes. We therefore globally identified the sets of Hic-5 blocked and non-blocked GBR and examined the chromatin remodeler requirements, the chromatin structure and the DNA sequence (specifying potential binding sites for other proteins) around these two classes of GBR. Our findings address the gene-specific characteristics that dictate binding site selection by transcription factors and determine the gene-specific actions of coregulators.

## Materials and methods

### Cell culture and siRNA transfection

U2OS osteosarcoma cells stably expressing wild-type GRα (U2OS-GRα) were a gift from Dr. Inez Rogatsky (Hospital for Special Surgery, New York, NY). U2OS cells were originally obtained from ATCC and stably transformed to express GRα [22]. The cells were authenticated by Short Tandem Repeat Profiling, tested negative for mycoplasma, and maintained as described [6]. Cells were grown in medium supplemented with 5% (vol/vol) FBS and transfected with siRNA using Lipofectamine RNAiMAX (Invitrogen). For double depletion of chromatin remodeler and Hic-5, equivalent amounts siRNA for a chromatin remodeler and Hic-5 (siHic5) were added. For single protein depletions, equivalent amounts of siRNA for the targeted protein and nonspecific control siRNA (siNS) were used such that the total volume and mass of siRNA was consistent. 48 h after siRNA transfection the cells were treated for the indicated length of time with 100 nM dex (Sigma) or an equivalent amount of ethanol as control. siRNA sequences for siNS, siHic-5, siBRM, and siCHD9 were previously described [16]

### Chromatin Immunoprecipitation followed by high-throughput sequencing

ChIP (Chromatin Immunoprecipitation) experiments were performed as previously described [16]. Briefly, U2OS-GRα cells grown on 15-cm dishes were transfected with the appropriate siRNAs. After 48 h, the cells were treated with 100 nM dex or equivalent amounts of ethanol for 1 h before crosslinking with 1% (v/v) formaldehyde for 10 min at room temperature and extracting chromatin from the harvested cells. Chromatin was sonicated 20–30 min (30 s on/off cycles) with the Biorupter (Diagenode) at 4°C to produce DNA fragment size of 400–600 bp. Immunoprecipitation of the sonicated chromatin samples was conducted with a cocktail of GR antibodies: H300 (6 μg, Santa Cruz Biotechnology), PA1-511A (2 μg, Thermo Scientific), D6H2L (2 μg, Cell Signaling Technology) in a volume of 1 ml containing chromatin from 20 x 10^6^ cells. Protein G Sepharose magnetic beads (GE Healthcare) were used to isolate the immune complexes with the crosslinked DNA. Purified DNA from the ChIP experiments were analyzed for quality using Agilent Technologies 2100 Bioanalyzer. Samples were submitted to Next-Generation Sequencing Core at the University of Southern California Norris Comprehensive Cancer Center for library preparation and sequencing. Single-end 75 bp DNA-sequencing data were generated for the samples using Illumina NEXTseq 500. Sequencing results produced 36–70 million raw reads per sample. After trimming the raw reads for quality and adapter sequence, the samples were mapped using BWA against the GRCh38/hg38 human reference genome [23]. 94–98% of the raw reads were mapped to the genome. Duplicate reads and reads mapping to mitochondrial DNA were removed. To determine the peaks with the MACS2+IDR method, mapped reads were analyzed using Model-based Analysis for ChIP-Seq version 2 (MACS2) [24] and the Irreproducibility Discovery Rate (IDR) framework developed for ENCODE [https://sites.google.com/site/anshulkundaje/projects/idr]. For peaks identified with the MACS2+DiffBind method, DiffBind [25] was used to determine the differentially bound peaks from the sets of peaks called by MACS2 in at least 2 samples from all of the conditions tested. False discovery rate adjusted p-value ≤ 0.01 and the indicated fold-change cut-off for the change in RPKM values for GR occupancy were used along with the DESeq2 option during DiffBind analyses to identify the set of differentially bound peaks and create the MA plots. Venn diagrams overlapping the different sets of peaks were created using ChIPpeakAnno [26]. Both DiffBind and ChIPpeakAnno are packages available on Bioconductor [27, 28]. The Integrative Genomics Viewer was used to visualize the ChIP-seq data [29]. Motif analysis was performed using Hypergeometric Optimization of Motif EnRichment (HOMER) with a 1 kb window centered at the GBR summit [30].

### Assay for Transposase-Accessible Chromatin using sequencing (ATAC-seq)

ATAC-seq was performed as previously described [31]. Briefly, nuclei were prepared from 50,000 U2OS-GRα cells and resuspended in the transposase reaction mix (FC-121-1030; Illumina). Transposition reaction was performed at 37°C for 30 min, and the samples were purified using a Qiagen PCR MinElute kit (28006; Qiagen). Library fragments were amplified using Nextera PCR Primers (FC-121-1011; Illumina) and NEBnext PCR master mix (0541; New England Lab) for a total of 6–8 cycles. The libraries were then purified and size selected in the 100–450 bp range using Agencourt AMPure XP (A63880; Beckman Coulter). The size-selected libraries were quantified using the Agilent Bioanalyzer. Samples were submitted to Next-Generation Sequencing Core at the University of Southern California Norris Comprehensive Cancer Center for sequencing. Paired-end 41 bp and 76 bp sequencing data were generated for the samples using Illumina NEXTseq 500. Sequencing results produced a total of 106–158 million raw reads per sample. Samples were mapped against the GRCh38/hg38 human reference genome using Bowtie 2 [32]. 96–98% of the raw reads mapped to the genome. Duplicated reads and reads aligned to mitochondrial DNA were removed. Accessible regions and peaks were identified using the broad peak calling parameters of MACS2. Chromatin accessibility profiles were created using the SeqPlots package [33] from Bioconductor by assessing the chromatin with ATAC-seq reads per million (RPM) per 10 nucleotides +/- 1kb from the GBR summit.

## Results

### Genome-wide analysis of GR binding regions that are blocked or not blocked by Hic-5

Previously we showed that CHD9 and BRM are required for the binding of GR to GR binding regions (GBR) associated with 2 representative *block* genes but not for GBR associated with 2 representative *ind* or *mod* genes [16]. Here we extended our previous studies by assessing whether the same site-specific chromatin remodeler requirement applies genome-wide. GR occupancy was assessed by ChIP-seq experiments with GR antibody, using U2OS-GRα osteosarcoma cells transfected with six different siRNA combinations: non-specific siRNA control (siNS/siNS), depletion of Hic-5 (siHic5/siNS), depletion of CHD9 (siCHD9/siNS), double depletion of Hic-5 and CHD9 (siCHD9/siHic5), depletion of BRM (siBRM/siNS), and double depletion of Hic-5 and BRM (siBRM/siHic5). Cells in each category were treated with 100 nM dex for 1 hour, and two biological replicates were performed on different days.

**Fig 1:**
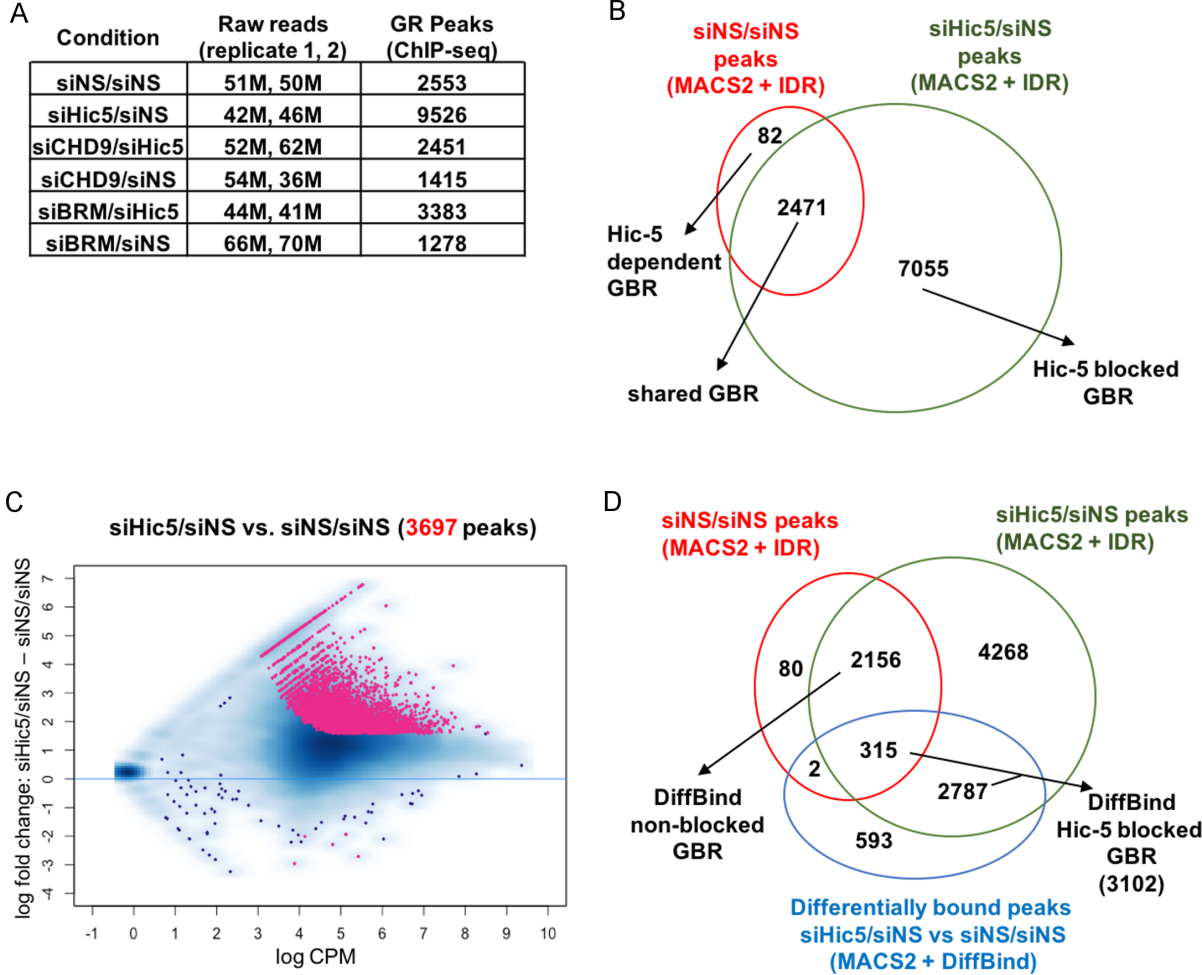
Identifying Hic-5 blocked GBR and non-blocked GBR. (A) Total GR peaks (or GR binding regions, GBR) identified for each condition using the MACS2+IDR method. ChIP-seq with GR antibody was performed on U2OS-GRα cells transfected with indicated siRNA combinations and treated with dex for 1 hr. Total raw reads for the duplicate samples are also shown. (B) Venn diagram showing overlap of GR peaks after 1 hr dex treatment in Hic-5 positive cells (siNS/siNS) and Hic-5 depleted cells (siHic5/siNS). (C) MA plot analysis to identify GBR with statistically altered level of GR binding between Hic-5 positive cells (siNS/siNS) and Hic-5 depleted cells (siHic5/siNS). X-axis shows the log_2_ of the average GR binding intensity for each GBR (in counts per million, cpm) from all conditions as mentioned in A. Y-axis shows the log_2_ fold-change in GR binding between siHic5/siNS and siNS/siNS for each GBR. Each red dot represents a GR peak that is significantly different between siHic5/siNS and siNS/siNS with FDR < 0.01 and at least a 3-fold change in binding. Blue dots represent peaks not differentially bound between siNS/siNS and siHic5/siNS with blue smears indicating overrepresentation of blue dots. (D) Identification of Hic-5 blocked and non-blocked GBR by statistical analysis of differential GR binding. Three-way Venn diagram overlapping GBR in Hic-5 positive and Hic-5 depleted cells identified by MACS2+IDR method with GBR having significantly different GR binding between these two conditions as defined by MACS2+DiffBind method. DiffBind Hic-5 blocked GR peaks are GBR found only in Hic-5 depleted cells using the MACS2+IDR method that have a significant difference in GR binding intensity between Hic-5 depleted cells and Hic-5 positive cells using the MACS2+DiffBind method. DiffBind non-blocked GBR are GR binding sites found in both Hic-5 positive and Hic-5 depleted cells using the MACS2+IDR method but have no significant difference in binding intensity between Hic-5 depleted cells and Hic-5 positive cells as defined by the MACS2+DiffBind method.

We first identified the sets of GBR that are blocked by Hic-5 (blocked GBR) or not blocked by Hic-5 (non-blocked GBR). For each condition, GBR were determined using the IDR framework with MACS2 as the peak caller (MACS2+IDR GBR) [24]. The number of GBR in cells depleted of Hic-5 (9526) was almost 4 times that in cells containing Hic-5 (2553), indicating that Hic-5 blocks GR binding to specific loci on a genome-wide basis (Fig 1A). When these 2 sets of GBR were overlapped, almost all of the GR peaks detected in cells containing Hic-5 were also detected in cells depleted of Hic-5 (2471 shared GBR), but there were 7055 Hic-5 blocked GBR that were called only after depletion of Hic-5 (Fig 1B).

In addition to analyzing GBR called by MACS2+IDR, we used a statistical method to compare quantitatively the GR occupancy at all GBR in cells containing or lacking Hic-5. We assessed the GR ChIP-seq data using peaks identified with default settings in MACS2 followed by statistical analysis of the peak intensities through DiffBind [25]. Our previous qPCR analysis of a few representative GBR [6, 16] revealed that GR ChIP signal increased upon Hic-5 depletion for most GBR, but the signal increased much more dramatically (more than 3-fold) for the block genes. Therefore, we established a quantitative cut-off of 3-fold along with a statistical cut-off (FDR < 0.01) for classification of Hic-5 blocked GBR versus non-blocked GBR. GBR were assessed by plotting the difference in log_2_ fold change of GR binding signal between cells depleted of Hic-5 and cells containing Hic-5 (y-axis) against the average binding intensity across all six experimental conditions (x-axis) (Fig 1C). Blue dots represent GBR that did not satisfy the statistical and quantitative cut-offs for changes caused by depletion of Hic-5, and blue smears indicate overrepresentation of blue dots. Red dots represent GBR that were significantly different (FDR < 0.01) and changed at least 3-fold in cells depleted of Hic-5. There was a general increase in GR binding intensity for all GBR upon Hic-5 depletion, as observed by the large clustering of dots above the log_2_ = 0 fold change line. Essentially all of the 3697 GBR that met the statistical and fold-change criteria had increased (rather than decreased) occupancy by GR in cells depleted of Hic-5. By overlapping the set of differentially bound GR peaks (3697 DiffBind GBRs, determined by MACS2+DiffBind, from Fig 1C) with the sets of MACS2+IDR-called GBR (from Fig 1A) in cells containing Hic-5 and depleted of Hic-5, we defined 3102 DiffBind Hic-5 blocked GBR and 2156 DiffBind non-blocked GBR (Fig 1D). Almost 90% of the blocked GBR (2787 peaks) were only observed in cells depleted of Hic-5; however, some of the blocked GBR (315 peaks) were called as peaks in cells containing or depleted of Hic-5 but had significantly different levels of GR occupancy (FDR < 0.01 and 3-fold change cutoff) in cells containing Hic-5 versus cells depleted of Hic-5.

### Differential requirement for chromatin remodelers CHD9 and BRM for GR occupancy at blocked versus non-blocked GBR

**Fig 2:**
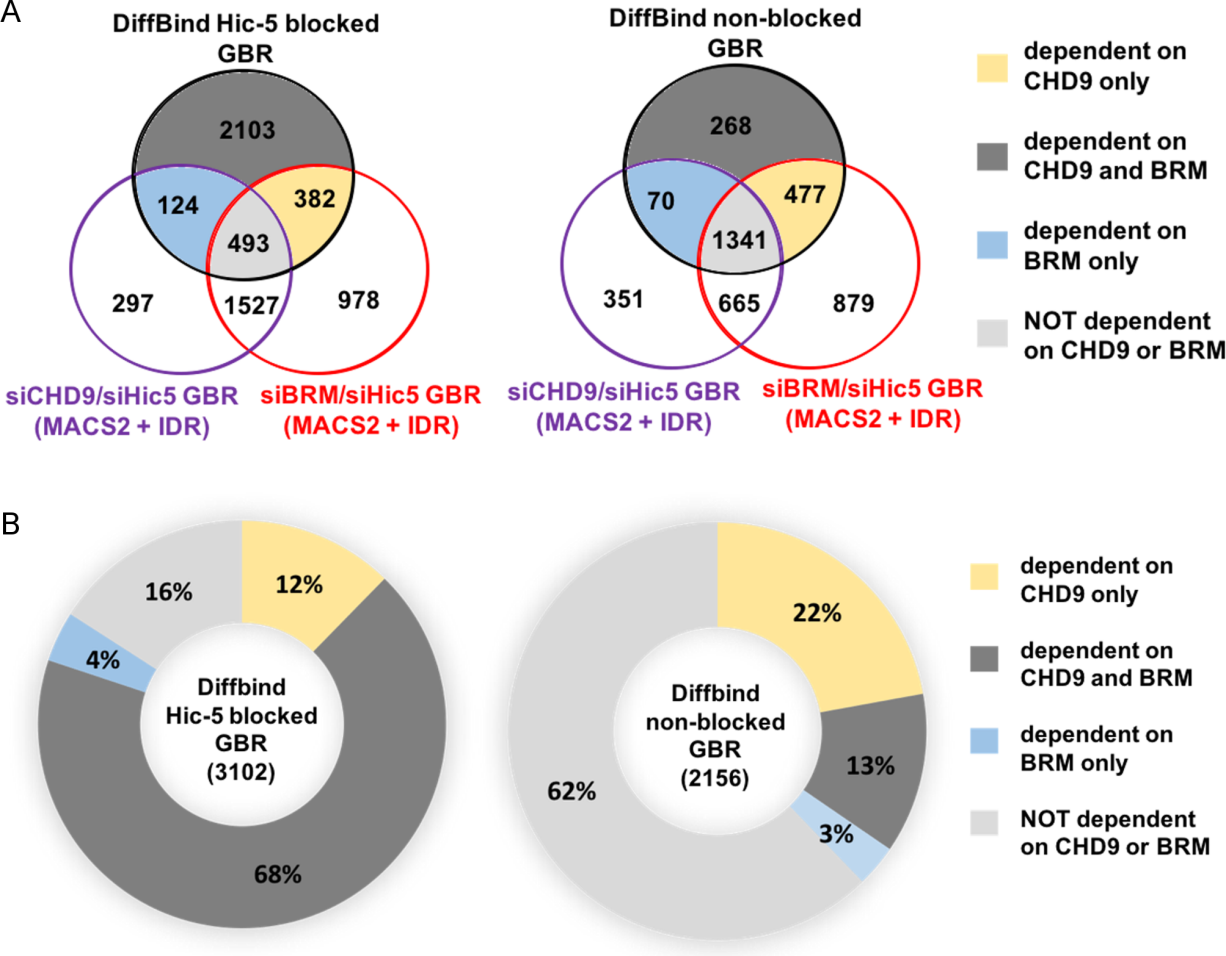
Hic-5 blocked GBR require CHD9 and BRM. Genome-wide GR ChIP-seq analysis was performed to evaluate the GR peaks dependent on CHD9 and BRM. (A) Three-way Venn diagram overlapping the following GBR sets: DiffBind Hic-5 blocked GBR (left) or non-blocked GBR (right) from Fig 1D; GBR in cells depleted of both CHD9 and Hic-5 (siCHD9/siHic5) from Fig 1A; and GBR in cells depleted of BRM and Hic-5 (siBRM/siHic5) from Fig 1A. Dark grey regions, GBR dependent on CHD9 and BRM; yellow regions, GBR dependent on CHD9, not BRM; blue regions, GBR dependent on BRM, not CHD9; light grey regions, GBR not dependent on CHD9 or BRM. (B) Pie charts showing percentage of blocked or non-blocked GBR that are dependent on CHD9 and/or BRM. The number of GBR in each colored compartment from A was divided by the total Hic-5 blocked GBR or non-blocked GBR to calculate percent of the whole.

We previously showed that CHD9 and BRM are required for GR occupancy at 2 blocked GBR but not at 4 non-blocked GBR [16]. Therefore, after defining the genome-wide sets of blocked and non-blocked GBR, we compared the requirement of these two sets of GBR for chromatin remodelers CHD9 and BRM. Since GR occupancy at the blocked GBR occurs only when Hic-5 is depleted, double depletion of a chromatin remodeler and Hic-5 is required to identify GBR that are dependent on the chromatin remodeler. The set of 3102 Hic-5 blocked GBR determined by the DiffBind analysis (from Fig 1D) was overlapped simultaneously with the MACS2+IDR-called GBR (from Fig 1A) from cells depleted of CHD9 and Hic-5 (siCHD9/siHic5) and from cells depleted of BRM and Hic5 (siBRM/siHic5) (Fig 2A, left side). Of the 3102 Hic-5 blocked GBR, 80% (382+2103 = 2485) were dependent on CHD9 for GR occupancy and 72% (124+2103 = 2227) were dependent on BRM (Fig 2A and 2B, left side). The CHD9 and BRM requirements were largely overlapping, such that 68% of the Hic-5 blocked peaks required both CHD9 and BRM for GR occupancy. In contrast, in a similar analysis of the 2156 non-blocked GBR, only 35% (477+268 = 745) and 16% (70+268 = 338) of the non-blocked peaks were dependent on CHD9 and BRM, respectively, for GR occupancy; only 13% of the non-blocked GBR required both CHD9 and BRM (Fig 2A and 2B, right side).

To conduct a more rigorous statistical analysis of blocked and non-blocked GBR that were dependent on chromatin remodelers CHD9 and BRM for GR occupancy, we first used the DiffBind method to identify CHD9-dependent GBR and BRM-dependent GBR. For each GBR the log_2_ fold change of GR binding signal between cells doubly depleted of Hic-5 and CHD9 and cells depleted of Hic-5 alone (y-axis) was plotted against the average binding intensity across all six experimental conditions (x-axis). In this analysis GR occupancy was significantly changed (FDR < 0.01, no fold change cut-off) for 3022 GBR due to depletion of CHD9; and 3903 GBR were differentially occupied due to depletion of BRM (Figure A in S1 Fig). The resulting sets of GBR for which GR occupancy is significantly dependent on CHD9 or BRM were overlapped with each other and with the set of DiffBind blocked GBR or DiffBind non-blocked GBR (Figure B in S1 Fig). In these overlaps, 43% (329+978 = 1307) of the 3102 peaks in the DiffBind blocked GBR required CHD9 and 59% (852+978 = 1830) were dependent on BRM (Figures B and C in S1 Fig). In contrast, 18% (136+261 = 397) and 38% (558+261 = 819) of the 2156 non-blocked DiffBind GR peaks were dependent on CHD9 and BRM, respectively. Thus, the requirement of CHD9 and BRM for GR occupancy is strongly associated with the GBR that are blocked with Hic-5 and not with the non-blocked GBR.

### Hic-5 blocked GBR are preferentially associated with Hic-5 blocked GR target genes

**Fig 3:**
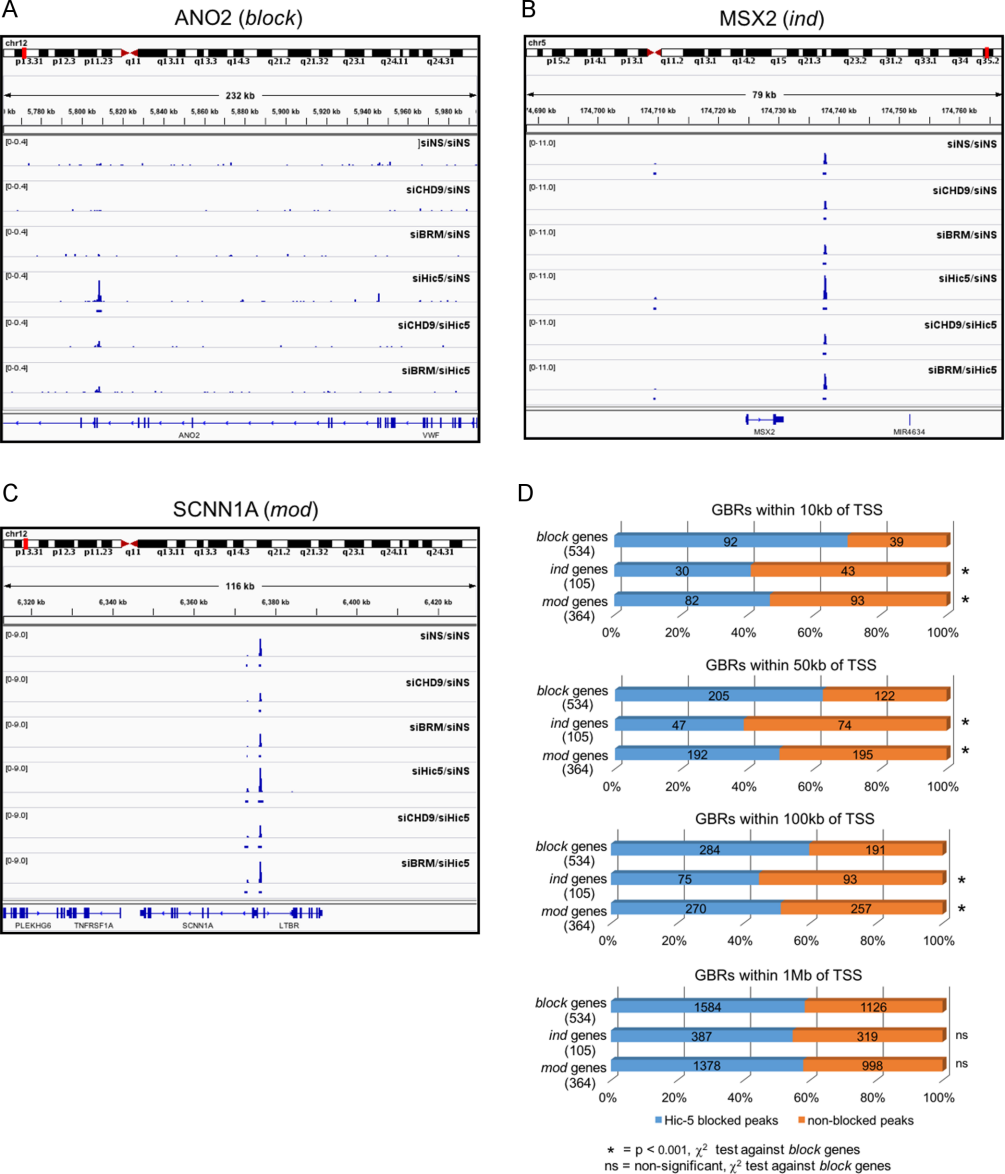
Hic-5 blocked GBR are preferentially enriched near the *block* genes. A-C) Integrative Genomics Viewer displaying the ChIP-seq track of GR occupancy near *block* gene ANO2 (A), *ind* gene MSX2 (B), and *mod* gene SCNN1A (C). D) Proportion of Hic-5 blocked GBR and non-blocked GBR within 10 kb, 50 kb, 100 kb and 1 Mb of TSS for all genes in each of the three gene classes. Blue bars represent the proportion of Hic-5 blocked GBR; orange bars represent the proportion of non-blocked GBR; the total number of GBR is indicated on each bar. χ^2^ test was performed to compare the ratio of blocked and non-blocked GBR near the *ind* genes and *mod* genes with the ratio of blocked and non-blocked GBR near the *block* genes. (* = p < 0.001, ns = not significant).

After defining the sets of Hic-5 blocked and non-blocked GBR, we determined the distribution of blocked and non-blocked GBR near the *block*, *ind*, and *mod* genes. From the ChIP-seq data, we first examined the GBR that are closest to representative genes from the *block*, *ind*, and *mod* gene classes. As we previously observed with ChIP-qPCR experiments on 2 representative block genes [6, 16], our current ChIP-seq data showed weak or undetectable GR occupancy at GBR nearest to the transcription start sites (TSS) of the representative *block* genes ANO2 and RP1L1 in cells containing Hic-5 and robust GR occupancy in cells depleted of Hic-5, such that MACS2+IDR only called the peak in cells depleted of Hic-5 (Fig 3A & Figure A in S2 Fig), as indicated by presence or absence of solid bars under the peaks. This phenotype is consistent with their classification as Hic-5 blocked GBR. After double depletion of CHD9 and Hic-5 or BRM and Hic-5, GR occupancy was reduced to a level that was not identified by the peak caller. In contrast, the GBR closest to representative *ind* genes MSX2 and IGFBP1 and representative *mod* genes SCNN1A and SPINK13 had called GR peaks in cells containing Hic-5 as well as in cells depleted of Hic-5 (Fig 3B and 3C; Figures B and C in S2 Fig), consistent with their classification as non-blocked GBR. GR occupancy at the GBR closest to these representative *ind* and *mod* genes was reduced by depletion of CHD9 or BRM, but the peaks were still called.

To assess genome-wide whether Hic-5 blocked GBR are preferentially enriched near the *block* genes compared with *ind* and *mod* genes, we determined the proportion of Hic-5 blocked GBR and non-blocked GBR within a specified distance of the TSS for all *block*, *ind*, and *mod* genes. The sets of *block*, *mod*, and *ind* genes used in our analysis were taken from our previously published RNA-seq data [16]. Within windows extending 10, 50, or 100 kb in either direction from the TSS of the 534 *block* genes, the percentages of GBR that were classified as Hic-5 blocked GBR were 67%, 60%, and 58%, respectively. In contrast, the corresponding percentages for the 105 *ind* genes were 39%, 37%, and 42%, and for the 364 *mod* genes the percentages were 44%, 47%, and 49% (Fig 3D). For each of these windows the percentages were significantly different (chi-square test) between *block* and *ind* and between *block* and *mod* gene sets. However, when the window was extended to 1 Mb from the TSS, the proportion of Hic-5 blocked to non-blocked GBR was similar across all gene classes (no significant differences), presumably due to the inclusion of many additional GBR that are not regulating the target genes. These data indicate that Hic-5 blocked GBR are preferentially associated with and enriched near the *block* gene class and not with the *ind* and *mod* gene classes.

### Chromatin structure at Hic-5 blocked GBR is less accessible than at non-blocked GBR in cells containing Hic-5

**Fig 4:**
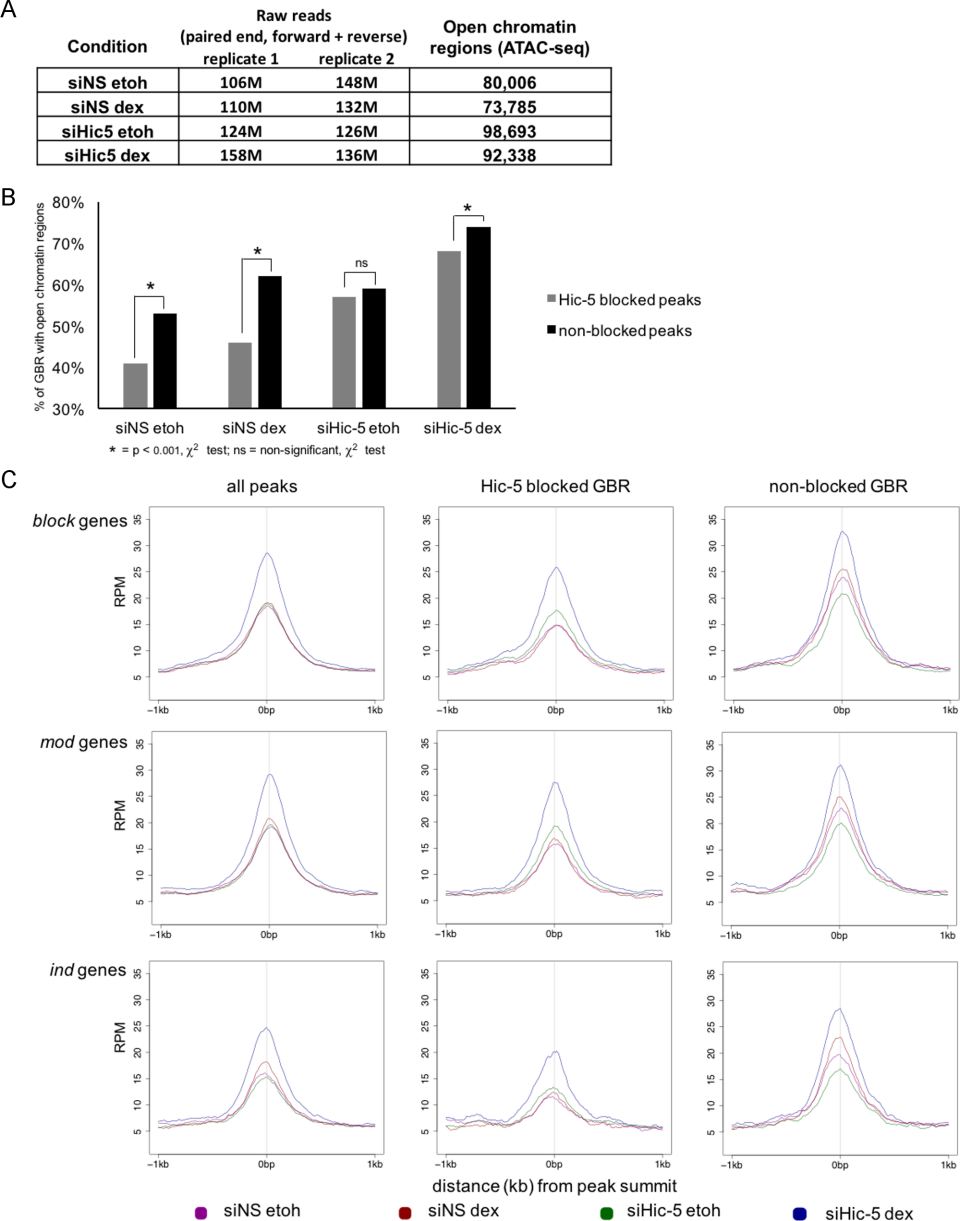
Chromatin at Hic-5 blocked GBR is less accessible than at non-blocked GBR. Genome-wide ATAC-seq analysis was performed to evaluate changes in chromatin structure caused by Hic-5 depletion in cells treated with dex or vehicle ethanol (etoh). (A) Total number of open chromatin regions from ATAC-seq analysis in cells containing or depleted of Hic-5 and treated with dex or vehicle ethanol. (B) Percentage of GBR with open chromatin in Hic-5 blocked GBR and non-blocked GBR for each condition described in A. χ^2^ significance test was performed to compare the proportion of Hic-5 blocked GBR with open chromatin and the proportion of non-blocked GBR with open chromatin for each condition. (* = p < 0.001, ns = not significant). (C) Average chromatin accessibility profile for all GBR near *block*, *mod* and *ind* genes. GBR within 100 kb of the TSS for all genes in each class were evaluated. Chromatin accessibility in reads per million (RPM) per 10 nucleotides was averaged for all GBR in the indicated GBR sets within a window extending 1 kb in either direction from the GBR peak summits in cells treated as follows: siNS etoh (purple), siNS dex (red), siHic-5 etoh (green), and siHic-5 dex (blue).

Since chromatin accessibility affects binding of GR and other transcription factors [16, 34], we assessed the chromatin accessibility at GBR associated with the three gene classes. Previous experiments using formaldehyde-assisted isolation of regulatory elements (FAIRE) followed by qPCR showed that chromatin accessibility at the GBR closest to the TSS of 3 representative *block* genes dramatically increased upon Hic-5 depletion and dex treatment [6, 16]. However, FAIRE-qPCR was not sensitive enough to detect changes in chromatin structure due to Hic-5 depletion in cells not treated with dex. Therefore, we performed Assay for Transposase-Accessible Chromatin using sequencing (ATAC-seq) to assess global chromatin accessibility in cells treated with dex or equivalent volume of vehicle ethanol, both in the presence and absence of Hic-5 (Fig 4A). In cells containing Hic-5 and treated with ethanol or dex, our analysis revealed 80,006 and 73,785 open chromatin regions, respectively. In cells depleted of Hic-5 and treated with ethanol or dex, we obtained 98,693 and 92,338 open chromatin regions, respectively. The ATAC-seq peaks were overlapped with the genome-wide sets of Hic-5 blocked and non-blocked GBR, as determined from the DiffBind ChIP-seq analyses (from Fig 1D), to assess chromatin accessibility at the GBRs. In cells containing Hic-5 (treated with either dex or ethanol) the percentage of Hic-5 blocked GBR with open chromatin (defined by ATAC-seq peaks called by MACS2+IDR) was significantly lower than the percentage of non-blocked GBR with open chromatin (Fig 4B). In cells depleted of Hic-5, the difference in the percentage of accessible blocked versus non-blocked GBR was less than in cells containing Hic-5 and was significant only in cells treated with dex.

We also examined the chromatin accessibility of the GBRs that lie within 100 kb of the TSS of the genes in each of the three gene classes. The average RPKM from ATAC-seq data, centered at the GBR summits were graphed to create an average chromatin accessibility profile of GBR for each of the four conditions tested by ATAC-seq: cells containing Hic-5 with or without dex (siNS etoh and siNS dex) and cells depleted of Hic-5 with or without dex (siHic-5 etoh and siHic-5 dex) (Fig 4C). Chromatin accessibility of blocked GBR associated with all three gene classes did not change between etoh and dex treatment in cells containing Hic-5, but in cells depleted of Hic-5 dex caused a robust increase in accessibility of the blocked GBR. In contrast, for the non-blocked GBR associated with all three gene classes, dex increased chromatin accessibility in the presence or absence of Hic-5. In general the chromatin accessibility of blocked GBR was less than the accessibility of non-blocked GBR; this was true in all four conditions tested (± Hic-5, ± dex) and for GBR near all three gene classes (*block*, *ind*, and *mod*). Of particular note was that the accessibility of blocked GBR in ethanol-treated cells was higher when Hic-5 was depleted than when Hic-5 was present; this was true for blocked GBR near all three gene classes. In contrast, the accessibility of the non-blocked GBR in ethanol treated cells was lower in Hic-5 depleted cells than in cells containing Hic-5. These data indicate that Hic-5 selectively interferes with the chromatin accessibility of blocked versus non-blocked GBR, both before dex treatment and after dex is added.

### ETS family binding motif is enriched near blocked GBR and near GBRs associated with *block* genes

**Fig 5:**
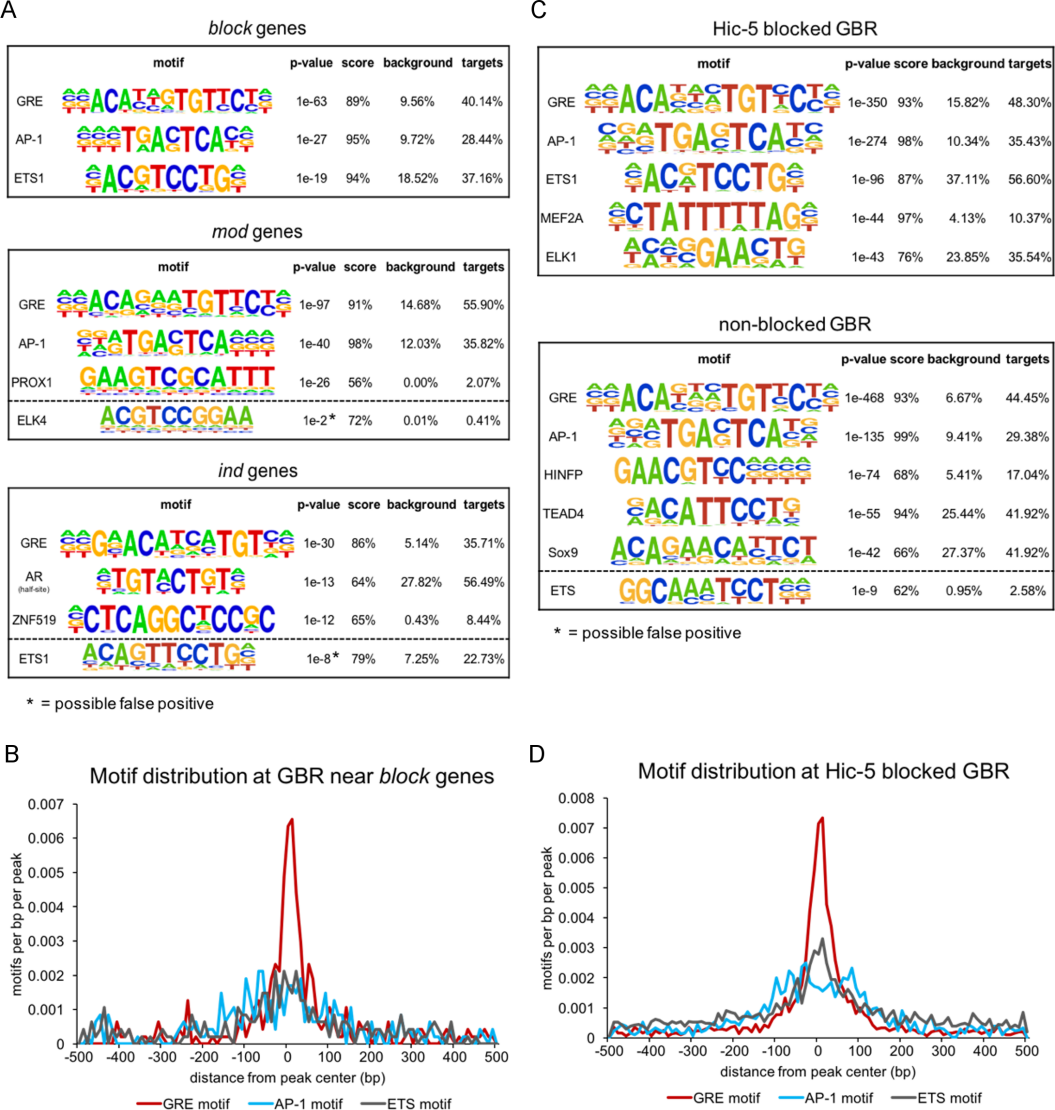
ETS motif is enriched at Hic-5 blocked GBR and GBR near the *block* genes. (A) ETS motif is enriched in GBR near *block* genes but not in GBR near *mod* and *ind* genes. *De novo* motif analysis was performed using HOMER; the top 3 ranked motifs are shown with their p-value, score for concordance of the *de novo* motif with the identified match, and prevalence near the GBR set examined. Motif analysis was performed in a 1-kb window centered on the GBR peak for all GBR within 100 kb of the TSS of the *block*, *mod* and *ind* genes. Motifs below the dotted lines are for the highest scoring member of the ETS family (shown for comparison to the *block* genes), which were not found as one of the top three motifs for *mod* and *ind* genes and indicated as possible false positives by HOMER. (B) GR, AP-1 and ETS motif distribution in a 1-kb window centered on the peak of all GBR near the *block* genes. Red, GRE motif; blue, AP-1 motif; black, ETS motif. (C) ETS motif is enriched at Hic-5 blocked GBR but not at non-blocked GBR. Motif analysis was performed, as in A, for all Hic-5 blocked and non-blocked GBR. The top 5 motifs are shown. ETS motif below the dotted line is shown for comparison, but was not one of the top 5 motifs and was indicated as a possible false positive by HOMER. (D) GR, AP-1 and ETS motif distribution near Hic-5 blocked GBR, presented as in B.

To explore further the characteristics of Hic-5 blocked versus non-blocked GBR, motif analyses were performed on different sets of GBR identified in this study. GBR within 100 kb of the TSS of all genes in each of the three gene classes were examined using *de novo* motif analysis with HOMER to identify enriched motifs in a 1-kb window centered on the GBR summits. Motifs were ranked by p-value, and the top three motifs for each gene class are shown (Fig 5A). A score indicating the degree of match between the *de novo* motif identified and the consensus sequence for the matched transcription factor is also shown, along with the percentage of GBR that contain this motif in the 1-kb window around the GBR peak. Beyond the top three motifs identified, the remaining motifs were found at less than 10% of the GBRs in each set examined. As expected, the most significant and most prevalent motif in all three gene classes matched closely to the glucocorticoid response element (GRE), which is the consensus binding sequence for GR. The next two top-ranked motifs were AP-1 and an ETS family motif for GBR near *block* genes, AP-1 and PROX1 for GBR near *mod* genes, and androgen receptor half-site and ZNF519 for *ind* genes. The ETS motif was present near 37% of the GBR associated with *block* genes, but it was not significantly enriched near GBR associated with *mod* and *ind* genes. When the distribution of the GRE, AP-1 and ETS motifs were examined relative to the center of a 1-kb window centered on the GBR peaks associated with the *block* genes, the GRE motif (as expected) was highly enriched at the center of the GBR peaks associated with *block* genes (Fig 5B). The ETS motif had a broader distribution within the 1-kb window but was also enriched near the center of the 1-kb window surrounding the GBR associated with the *block* genes. The same was true for the AP-1 motif, but it was more dispersed from the summit of the GBR peaks than the ETS motif.

When *de novo* motif analysis was also conducted for all of the 3102 DiffBind Hic-5 blocked GBR and for all of the 2156 DiffBind non-blocked GBR (from Fig 1D), the ETS motif was significantly associated with the blocked GBR and was found near the majority of the blocked GBR (Fig 5C). In contrast, a sequence resembling the ETS protein family binding motif was found near less than 3% of the non-blocked GBR and was not significantly enriched. As observed with the *block* genes (Fig 5B), the ETS motifs near the 3102 blocked GBR were broadly distributed within the 1-kb window around the center of the GBR but were enriched near the center of the GBR, and the AP-1 motifs were more broadly dispersed from the center of the GBR peak (Fig 5D). When we measured the mean distances of the GRE, ETS, and AP-1 motifs from the summits of the GBR, we found that distribution of the GRE motifs was closer to the center of the GBR than the other two motifs (Figure A in S3 Fig).

To look for indications of possible functional relationships among the three motifs, we examined the subsets of all Hic-5 blocked GBR that contain GRE, ETS and AP-1 motifs (Figure B in S3 Fig). Among the 3102 DiffBind Hic-5 blocked GBR, 77% contained at least 1 of these motifs; 44% had a GRE, 52% had an ETS motif, and 32% had an AP-1 motif. Of the GBR with an AP-1 motif, 51% also had an ETS motif, but only 35% of GBR with an ETS motif also had an AP-1 motif (Figure C in S3 Fig). When we separately examined blocked GBR that required CHD9 versus BRM, we found very similar occurrence of GRE, AP-1 and ETS motifs (Figure D in S3 Fig).

*De novo* motif analysis was also performed for the sets of Hic-5 blocked and non-blocked GBR that became newly accessible (by called ATAC-seq peaks) upon depletion of Hic-5 (S4 Fig, the shaded sector of the Venn diagrams). A *de novo* motif closely resembling an ETS consensus binding site was significantly enriched within a 1-kb window centered on the peaks of the blocked GBR that became newly accessible upon Hic-5 depletion in cells treated with dex or vehicle ethanol (Figure A and B in S4 Fig). However, motifs resembling ETS binding sites were not significantly enriched near non-blocked GBR that became newly accessible upon Hic-5 depletion (Figure C and D in S4 Fig). Furthermore, the percentage of blocked GBR that became newly accessible upon Hic-5 depletion (15% in ethanol-treated cells and 22% in dex-treated cells), was almost twice that for the non-blocked GBR (8% in ethanol-treated cells and 13% in dex-treated cells). Thus, the ETS motif was found to be significantly associated with *block* genes and with blocked GBR, but not with non-blocked GBR or with *ind* or *mod* genes.

## Discussion

Our results demonstrate that Hic-5 has a major influence on binding site selection by GR. The number of GBR detected by a simple peak calling analysis nearly quadrupled upon Hic-5 depletion (Fig 1A), while a DiffBind analysis of sites with significantly enhanced GR binding upon Hic-5 depletion found a doubling of the number of GBR when Hic-5 was depleted (Fig 1C and 1D). By integrating this information on Hic-5 effects on GR binding at specific GBR with an analysis of chromatin remodeler requirements, chromatin conformation analysis by ATAC-seq, and previously obtained RNA-seq data [16], we defined specific characteristics that distinguish the Hic-5 blocked GBR from the non-blocked GBR.

### Hic-5 blocked GBR are preferentially associated with *block* genes, compared with *ind* and *mod* genes

We examined the GBR located within various distances of the TSS of all *block*, *ind*, and *mod* genes to determine the relative prevalence of Hic-5 blocked and non-blocked GBR near genes in each of the three gene classes. There was an enrichment of Hic-5 blocked GBR near the *block* genes compared to the *ind* and *mod* genes. 67% of the GBR located within 10 kb of any *block* gene TSS were Hic-5 blocked GBR, compared to 39% and 44% for *ind* and *mod* genes, respectively, and the ratio of blocked to non-blocked GBR near the *block* genes was significantly different from the ratios for the *ind* and *mod* genes (Fig 3D). Similar results were found when the distance from the TSS was increased to 50 kb or 100 kb. In contrast, when the distance from the TSS was increased to 1 Mb, the ratios of blocked to non-blocked GBR were indistinguishable between the three gene classes, indicating that the blocked GBR appear to be preferentially concentrated near the *block* genes. The enrichment of Hic-5 blocked GBR, and hence, GBR that require CHD9 and BRM for GR occupancy, distinguishes the GBR near the *block* genes from the GBR near *ind* and *mod* genes. In spite of the correlative nature of the data linking blocked GBR with the *block* gene class, the preferential co-localization of Hic-5 blocked GBR with genes that are blocked by Hic-5 from responding transcriptionally to dex suggests the logical conclusion that the blocked GBR are responsible for mediating the dex regulation of the *block* class of genes, while the non-blocked GBR are the regulatory sites responsible for dex regulation of the *ind* and *mod* genes.

### Chromatin remodelers CHD9 and BRM are preferentially required for Hic-5 blocked GBR versus non-blocked GBR

Our previous study showed that for the 2 GBR that are the closest to 2 selected *block* genes, Hic-5 blocks GR binding, and after Hic-5 depletion CHD9 and BRM are required for robust GR binding [16]. In contrast Hic-5 did not block binding to the most proximal GBR to 2 *ind* genes and 2 *mod* genes, and CHD9 and BRM were not required for GR binding to those GBR [16]. In the current study we found the same pattern genome-wide. By a peak calling analysis 84% of Hic-5 blocked GBR required CHD9 or BRM, and 68% required both of them for GR binding; on the other hand, only 38% of non-blocked GBR required CHD9 or BRM and only 13% required both (Fig 2B). When the same question was examined by a statistical method (DiffBind) to assess significant changes in GR binding, 70% of blocked Hic-5 GBR required CHD9 or BRM (32% required both), while only 44% of non-blocked GBR required either CHD9 or BRM (12% required both). Previous studies have shown that chromatin remodeling enzymes work in a coordinated fashion with GR to remodel the chromatin and allow GR to bind [16, 17, 19, 35, 36]. Thus, a distinguishing feature of Hic-5 blocked GBR genome-wide is their requirement for CHD9 and BRM. We previously showed that Hic-5 actually prevents GR binding to CHD9 and BRM but not to other chromatin remodelers such as BRG1 [16]. Since it was previously demonstrated that many if not most DNase I hypersensitive sites require more than one chromatin remodeler to maintain their chromatin accessibility and different hypersensitive sites require different combinations of chromatin remodelers [34], our findings of differential requirements for CHD9 and BRM explain mechanistically why Hic-5 blocks only a specific subset of GBR. The large overlap of the CHD9 and BRM requirement among Hic-5 blocked GBR suggests that they may act cooperatively to remodel the GBR, with each chromatin remodeler contributing specific remodeling actions.

The results support our hypothesis that gene-specific actions of Hic-5 depend on the gene-specific chromatin remodeler requirements, with *block* genes requiring CHD9 and BRM for GR binding to the GBR, but not *ind* or *mod* genes. Site-specific action of the chromatin remodeling enzymes has also been observed in other studies [34, 36]. The site-specific chromatin remodeler requirements support the idea that CHD9 and BRM regulate specific pathways of glucocorticoid function [16].

### Effects of Hic-5 on chromatin conformation at Hic-5 blocked and non-blocked GBR

Chromatin structure plays an important role in the binding of GR and other transcription factors to DNA [21, 37, 38]. Genome-wide studies of GR binding and chromatin accessibility, as measured by DNAse I hypersensitivity, have shown that GR binding mainly occurs at chromatin accessible sites that exist prior to hormone treatment [38]. However, upon GR activation the preexisting accessible GR binding sites can undergo additional chromatin remodeling [6, 39]. Additionally, studies using the MMTV promoter as a model system showed that GR was able to bind relatively inaccessible chromatin by triggering localized chromatin remodeling with the assistance of ATP-dependent chromatin remodelers [40].

In the current study, we used ATAC-seq to show that the number of accessible chromatin sites genome-wide (identified as called ATAC-seq peaks) increased when Hic-5 was depleted (Fig 4A) indicating a global effect of Hic-5 on many chromatin sites, not just at GBR. Thus, Hic-5 may restrict the binding site selection of other transcription factors in addition to GR. To specifically focus on GBR, ATAC-seq and GR ChIP-seq data were overlapped, revealing that the fraction of Hic-5 blocked GBR that are chromatin accessible is lower than the fraction of chromatin accessible non-blocked GBR (Fig 4B). Chromatin accessibility around Hic-5 blocked GBR was increased by dex only in cells depleted of Hic-5, while chromatin accessibility at non-blocked GBR was increased by dex in cells containing or depleted of Hic-5 (Fig 4C). This difference in blocked GBR and non-blocked GBR was observed for GBR near all three gene classes. In addition, Hic-5 depletion also slightly enhanced ATAC-seq signals at Hic-5 blocked GBR before dex treatment; in contrast, the ATAC-seq signal for non-blocked GBR decreased slightly in cells depleted of Hic-5 and not treated with dex (Fig 4C). Again, this pattern was observed for GBR near all three gene classes. Our results indicate a major effect of Hic-5 on chromatin remodeling of blocked GBR in response to dex and a minor effect on the basal (pre-dex) chromatin structure of blocked GBR, suggesting that both of these mechanisms contribute to the overall effect of Hic-5 on the response of blocked GBR to dex. The differential requirement for CHD9 and BRM to allow GR binding at blocked versus non-blocked GBR provides a likely molecular mechanism for the post-dex effect of Hic-5, with Hic-5 preventing GR from recruiting CHD9 and BRM to open the chromatin conformation and hence allow more robust GR binding. The specific mechanism for the pre-dex effect presumably depends on some other characteristic of blocked versus non-blocked GBR, such as less accessible chromatin conformation (Fig 4B) or different occupancy by other transcription factors at blocked versus non-blocked GBR, as suggested by motif analysis (Fig 5). This issue is explored below.

### ETS family binding motifs are prevalent near blocked GBR

GR binds directly to DNA as a homodimer, with a consensus 15-bp motif composed of two pseudopalindromic 6 base pair half-sites separated by a 3 base pair spacer [41]. GREs were the most significant and most prevalent motif for the GBR in all three gene classes, and for blocked and non-blocked GBR genome-wide (Fig 5A and 5C). The specific nucleotide sequence of the GR binding site can affect GR conformation and function [37, 42, 43], but our *de novo* motif analysis did not find any obvious sequence differences for GREs associated with different gene classes or for blocked versus non-blocked GBR. GR can also bind indirectly to specific DNA sites by binding to other transcription factors that are directly binding to DNA [44–46], and in general approximately half of the GBRs in each of the various GBR subsets that we examined contained a GRE-like motif. One commonly found motif associated with GBR is the AP-1 motif. AP-1 has been shown to maintain an accessible chromatin conformation that facilitates GR access to the GBR [47]. Indeed, the AP-1 motif was significantly associated with a high percentage of blocked and non-blocked GBR. In comparing motifs associated with blocked versus non-blocked GBR, the only motif our analysis found with a strong differential enrichment was an ETS family binding motif. This motif was significantly enriched and highly prevalent near blocked but not non-blocked GBR (Fig 5C) and was also significantly enriched and highly prevalent near GBR associated with *block* genes but not GBR associated with *ind* and *mod* genes (Fig 5A). The best match for the ETS family motif identified by HOMER was with the ETS1 family member, but the motifs for other ETS family members are also similar to the ETS1 motif [48, 49], so it remains to be determined exactly which ETS protein(s) may bind these motifs. ETS1 has previously been implicated in tumor progression [50], immunity [51], and angiogenesis [52]. ETS1 interacts with various other transcription factors including RUNX2 [53], STAT5 [54], and AP-1 [55]. ETS family member ETS2 interacts physically and functionally with GR [56], and a previous study also found an ETS family motif enriched near GR binding sites [57]. In future experiments it will be interesting to examine what role ETS family members may play in Hic-5 dependent blocking of GR binding and chromatin remodeling at blocked GBR.

Among many possible mechanisms by which ETS factors could contribute to the blocked GBR phenotype, we mention a few ideas here. Since ETS factors can initiate chromatin remodeling [58] and serve as pioneer factors for androgen receptor [59], we speculate that ETS factors could promote GR binding at Hic-5 blocked GBR, but require CHD9 and/or BRM in order to create open chromatin for GR binding. Alternatively, ETS factors could recruit Hic-5, which blocks GR binding by interfering with the necessary chromatin remodeling by CHD9 and BRM. The frequent occurrence of AP-1 motifs in addition to ETS motifs near blocked GBR suggests another possibility, that AP-1 and ETS factors could somehow cooperate to promote the Hic-5 blocked phenotype of at least some blocked GBRs. However, even if an AP-1 type protein is cooperating with an ETS factor at some blocked GBR, there must also be other mechanisms independent of AP-1 sites, since many blocked GBR lack nearby AP-1 motifs and only 35% of blocked GBR containing an ETS motif also have an AP-1 motif (S3 Fig).

The results presented here strongly suggest that chromatin remodeler requirements, chromatin structure and the DNA sequence contribute to the gene-specific actions of Hic-5 on the binding of GR and remodeling of chromatin at GBR. The GBR near the *block* genes, but not the *mod* or *ind* genes, required CHD9 and BRM for GR occupancy and dex-induced chromatin remodeling; blocked GBR were less chromatin accessible than the non-blocked GBR; and DNA motif analysis revealed an ETS family motif enriched at blocked GBR and GBR near the *block* genes but not at non-blocked GBR or GBR near the *ind* and *mod* genes. Hic-5 has been shown to block occupancy of other transcription factors, such as the estrogen receptor α, in a site-specific manner and prevent hormone-induced expression of a subset of estrogen receptor target genes [60]. Hic-5 also serves as a coregulator for a number of other transcription factors [9–15]. Furthermore, several other coregulators promote hormonal regulation of some GR target genes while blocking regulation of another subset of GC regulated genes in A549 cells [5]. Thus, the mechanism we document for the gene-specific actions of GR and Hic-5 − involving chromatin environment, differential requirements for chromatin remodelers, binding of nearby proteins, and DNA sequence − is likely to be more broadly applicable. Although our study focuses on GR and Hic-5, binding site selection and gene-specific actions of coregulators are important components of transcriptional regulation for all transcription factors. Our study elucidates important aspects of the mechanisms responsible for the gene-specific requirements for coregulators that control transcription.

## Supporting information

S1 Fig Differential GR peak analysis shows Hic-5 blocked GBR that require CHD9 and BRM. Hic-5 blocked and non-blocked GBR were evaluated genome-wide with DiffBind to identify GBR with significantly different GR occupancy in cells containing or depleted of CHD9 and BRM. (A) MA plot analysis to identify GR peaks with statistically altered level of GR binding between cells depleted of Hic-5 only (siHic5/siNS) and cells doubly depleted of CHD9 and Hic-5 (left side) or doubly depleted of BRM and Hic-5 (right side). For each GBR the log_2_ fold-change in GR binding between siCHD9/siHic5 and siHic5/siNS samples or between siBRM/Hic5 and siHic5/siNS samples (Y-axis) is plotted against the log_2_ of the average GR binding intensity from all conditions tested (cpm). Each red dot represents a differential GR peak with significantly altered GR binding (FDR < 0.01) between the doubly depleted and Hic-5 depleted samples. Blue dots represent peaks not differentially bound between the indicated conditions with blue smears demonstrating overrepresentation of blue dots. (B) Three way Venn diagrams overlapping the following GBR sets: DiffBind Hic-5 blocked GBR (left side) or non-blocked GBR (right side); the set of GBR that had significantly reduced GR occupancy when CHD9 and Hic-5 were depleted compared with depletion of Hic-5 alone; and the set of GBR that had significantly reduced GR occupancy when BRM and Hic-5 were depleted compared with depletion of Hic-5 alone. Yellow regions, GBR dependent on CHD9; blue regions, GBR dependent on BRM; dark gray regions, GBR dependent on both CHD9 and BRM; light grey regions, GBR not dependent on CHD9 or BRM. (C) Pie charts showing percentage of blocked and non-blocked GBR that are dependent on CHD9 and/or BRM. The number of GBR in each colored compartment from B was divided by the total Hic-5 blocked GBR or non-blocked GBR to calculate percent of the whole.
(TIF)

S2 Fig GR ChIP-seq signals near a representative gene for each of the three gene classes. ChIP-seq Integrative Genome Viewer display of GR occupancy near *block* gene RP1L1 (A), *ind* gene IGFBP1 (B), and *mod* gene SPINK13 (C).
(TIF)

S3 Fig Analysis of GRE, AP-1 and ETS motifs for Hic-5 blocked GBR. (A) Mean distance and standard deviation of the GRE, AP-1, and ETS motifs from the center of the GBR in base pairs (bp). (B) Venn diagram overlapping Hic-5 blocked GBR containing the GRE motif, AP-1 motif, or ETS motif. (C) Hic-5 blocked GBR with each motif that also includes one of the other two motifs. Percentages were calculated with the numbers in B. (D) Motif analysis of Hic-5 blocked GBR that are dependent on CHD9 or on BRM. *De novo* motif analysis was performed using HOMER; the top 5 ranked motifs are shown with their p-value, score for concordance of the *de novo* motif with the identified match, and prevalence near the GBR set examined.
(TIF)

S4 Fig ETS family motif is enriched at Hic-5 blocked GBR that become newly chromatin-accessible when Hic-5 is depleted. Hic-5 blocked GBR (A-B) or non-blocked GBR (C-D) were overlapped with two other sets: open chromatin regions identified by ATAC-seq in Hic-5 positive cells; and open chromatin regions in Hic-5 depleted cells. This was done for ATAC-seq data from cells treated with vehicle ethanol (etoh, A and C) or cells treated with dex (C and D). The shaded region of each three-way Venn diagram indicates the GBR that become newly chromatin accessible upon Hic-5 depletion. *De novo* motif analysis was performed on the GBR in each shaded region, using HOMER. The top 3 ranked motifs are shown with their p-value, score for concordance of the *de novo* motif with the consensus sequence of the identified match, and prevalence near the GBR in the set examined. Motif analysis was performed in a 1-kb window centered on the GBR peak for all GBR belonging to the set examined. Motifs below the dotted lines in C and D (shown for comparison to A and B) are for the highest scoring member of the ETS family, which were not one of the top three motifs and were indicated as possible false positives in C and D by HOMER.
(TIF)

S1 Dataset ChIP-seq peaks called by MACS2+IDR. The dataset contains the ChIP sequencing data for GR with consensus called peaks using the MACS2+IDR method for each condition from Fig 1A, with peaks from each condition described in separate sheets of the Excel file. Within each sheet: column A, chromosome number; column B, chromosome start position; column C, chromosome end position; column D, average enrichment under the peak in counts per million (CPM); column E, −log10 of the p-values for each peak; column F, −log10 of the FDR adjusted p-values (q-values).
(XLSX)

S2 Dataset Defining GBR with significantly different GR occupancy when Hic-5, CHD9, or BRM is depleted. The dataset contains data from three comparisons of ChIP-seq peak sets by the MACS2+DiffBind method: sheet 1 (illustrated in Fig 1C and 1D) compares siHic5siNS vs. siNSsiNS to determine the set of differentially bound GBR between cells containing Hic-5 and cells depleted of Hic-5; sheet 2 (illustrated in S1 Fig) compares siCHD9siHic5 vs. siHic5siNS to determine the differentially bound GBR between Hic-5 depleted cells without CHD9 and Hic-5 depleted cells with CHD9; sheet 3 (also illustrated in S1 Fig) compares siBRMsiHic5 vs. siHic5siNS to determine the differentially bound GBR between Hic-5 depleted cells without BRM and Hic-5 depleted cells with BRM. For the first comparison (siHic5siNS vs. siNSsiNS), 3-fold change cut off and FDR value < 0.01 were applied. For other comparisons (siCHD9siHic5 vs. siHic5siNS and siBRMsiHic5 vs. siHic5siNS) only FDR value < 0.01 was applied. Column A, chromosome number; column B, chromosome start position; column C, chromosome end position; column D, average enrichment under the peak in counts per million (CPM); column E, fold change for the comparison; column F, p-values; column G, FDR adjusted p-values.
(XLSX)

S3 Dataset Hic-5 blocked and non-blocked GBR. This dataset contains the Hic-5 blocked and non-blocked GBR with chromosome positions for each peak. Sheet 1–3 are the sets of GBR obtained by overlapping the siHic5siNS peaks with the siNSsiNS peaks identified with the MACS2+IDR method (illustrated in Fig 1B). Sheet 1 contains the Hic-5 blocked GBR, sheet 2 is the shared GBR, and sheet 3 is the peaks for the Hic-5 dependent GBR. Sheets 4 and 5 are the Hic-5 blocked GBR and non-blocked GBR, respectively, when differentially bound peaks between siHic5siNS and siNSsiNS as defined by DiffBind are overlapped with the sets of peaks identified using the MACS2+IDR (illustrated in Fig 1D). Within each sheet of the Excel file: column A, chromosome number; column B, chromosome start position; column C, chromosome end position.
(XLSX)

S4 Dataset Open chromatin regions defined by ATAC-seq. The dataset contains the ATAC sequencing data of open chromatin regions as identified by MACS2 (illustrated in Fig 4A), with peaks for each condition described in separate sheets of the Excel file: ethanol treated cells containing Hic-5 (siNS etoh), dex treated cells containing Hic-5 (siNS dex), ethanol treated cells depleted of Hic-5 (siHic5 etoh), dex treated cells depleted of Hic-5 (siHic5 dex). Consensus peaks between the two replicates for each condition are shown. Within each sheet: column A, chromosome number; column B, chromosome start position; column C, chromosome end position; column D, average enrichment under the peak in counts per million (CPM); column E, −log10 of the p-values for each peak; column F, −log10 of the FDR adjusted p-values (q-values).
(XLSX)
