# Supplementary figures and images for: Different chromatin and DNA sequence characteristics define glucocorticoid receptor binding sites that are blocked or not blocked by coregulator Hic-5
Source: PLoS One. 2018 May 8;13(5):e0196965. doi: 10.1371/journal.pone.0196965 (PMC5940187; doi:10.1371/journal.pone.0196965)

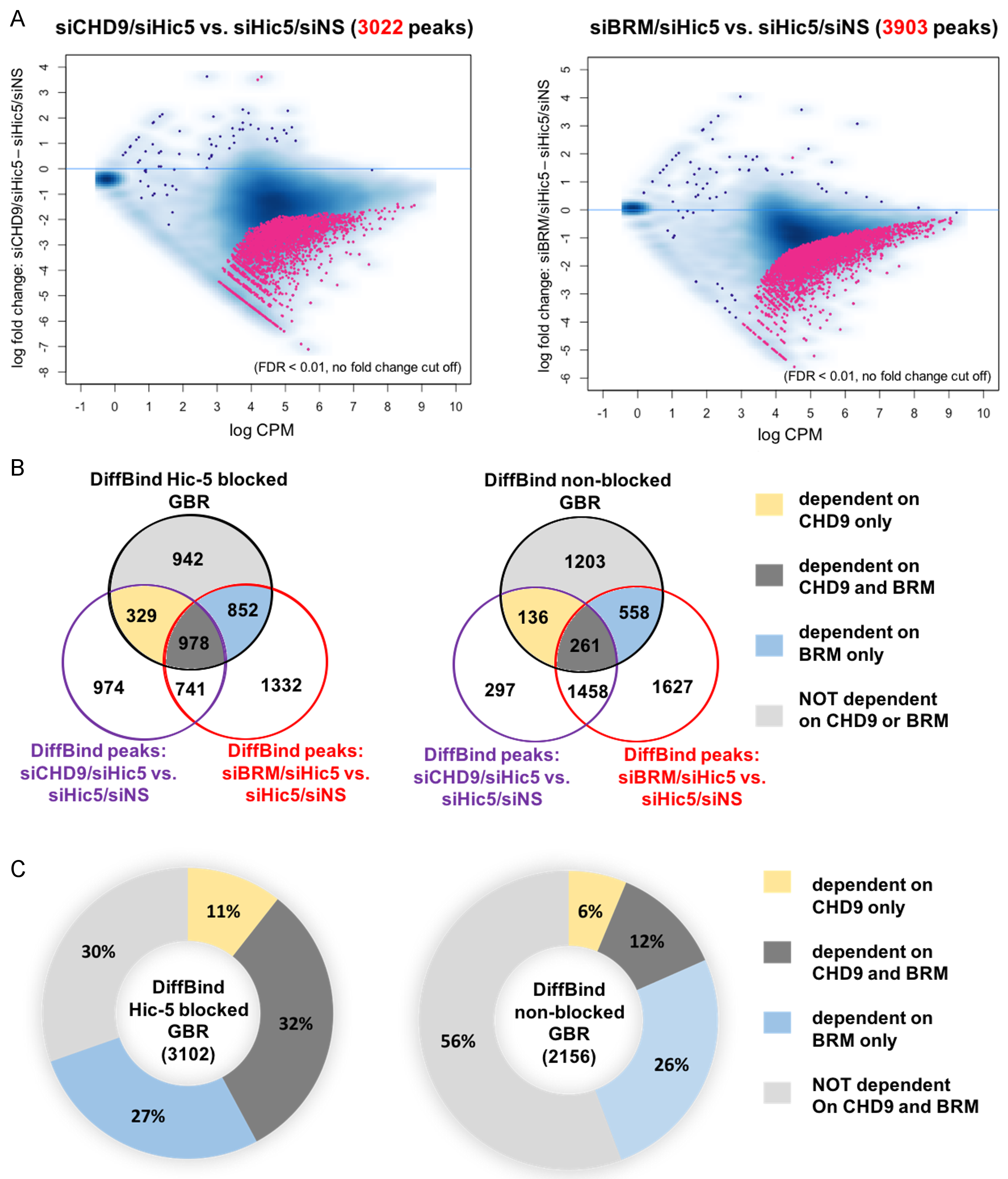

Supplement: S1 Fig — Hic-5 blocked and non-blocked GBR were evaluated genome-wide with DiffBind to identify GBR with significantly different GR occupancy in cells containing or depleted of CHD9 and BRM. (A) MA plot analysis to identify GR peaks with statistically altered level of GR binding between cells depleted of Hic-5 only (siHic5/siNS) and cells doubly depleted of CHD9 and Hic-5 (left side) or doubly depleted of BRM and Hic-5 (right side). For each GBR the log2 fold-change in GR binding between siCHD9/siHic5 and siHic5/siNS samples or between siBRM/Hic5 and siHic5/siNS samples (Y-axis) is plotted against the log2 of the average GR binding intensity from all conditions tested (cpm). Each red dot represents a differential GR peak with significantly altered GR binding (FDR < 0.01) between the doubly depleted and Hic-5 depleted samples. Blue dots represent peaks not differentially bound between the indicated conditions with blue smears demonstrating overrepresentation of blue dots. (B) Three way Venn diagrams overlapping the following GBR sets: DiffBind Hic-5 blocked GBR (left side) or non-blocked GBR (right side); the set of GBR that had significantly reduced GR occupancy when CHD9 and Hic-5 were depleted compared with depletion of Hic-5 alone; and the set of GBR that had significantly reduced GR occupancy when BRM and Hic-5 were depleted compared with depletion of Hic-5 alone. Yellow regions, GBR dependent on CHD9; blue regions, GBR dependent on BRM; dark gray regions, GBR dependent on both CHD9 and BRM; light grey regions, GBR not dependent on CHD9 or BRM. (C) Pie charts showing percentage of blocked and non-blocked GBR that are dependent on CHD9 and/or BRM. The number of GBR in each colored compartment from B was divided by the total Hic-5 blocked GBR or non-blocked GBR to calculate percent of the whole. (TIF) [file pone.0196965.s001.tif]

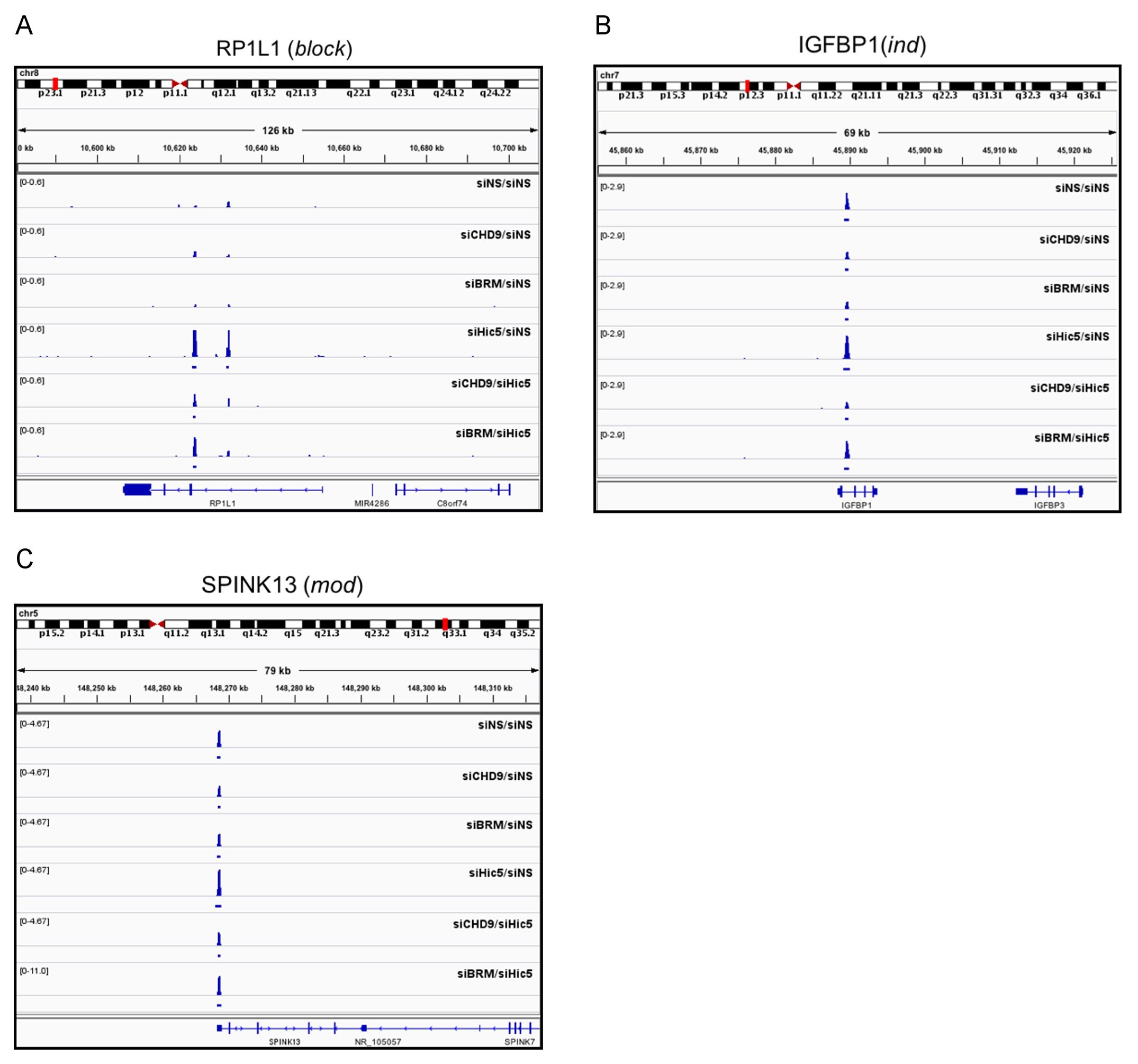

Supplement: S2 Fig — ChIP-seq Integrative Genome Viewer display of GR occupancy near block gene RP1L1 (A), ind gene IGFBP1 (B), and mod gene SPINK13 (C). (TIF) [file pone.0196965.s002.tif]

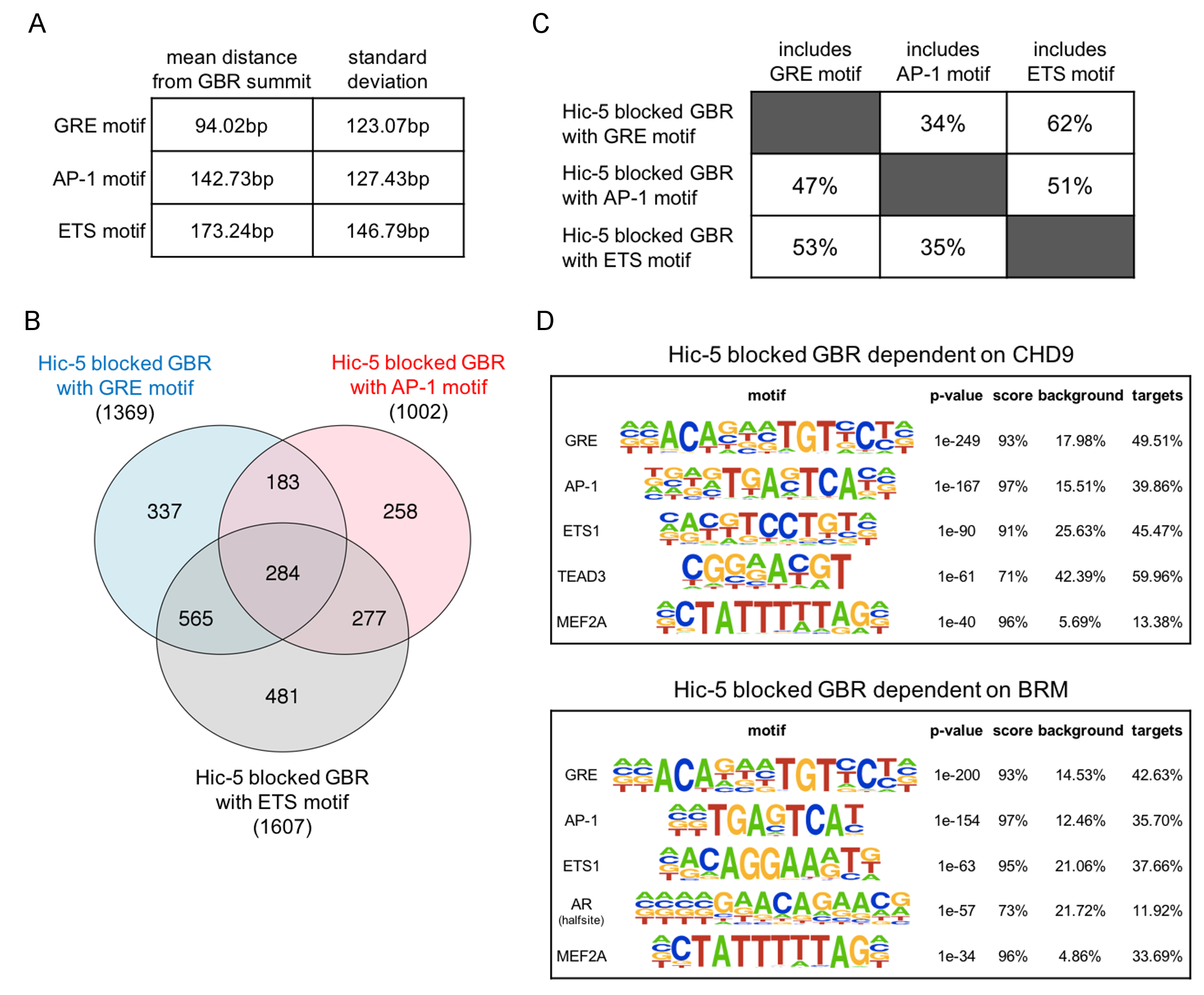

Supplement: S3 Fig — (A) Mean distance and standard deviation of the GRE, AP-1, and ETS motifs from the center of the GBR in base pairs (bp). (B) Venn diagram overlapping Hic-5 blocked GBR containing the GRE motif, AP-1 motif, or ETS motif. (C) Hic-5 blocked GBR with each motif that also includes one of the other two motifs. Percentages were calculated with the numbers in B. (D) Motif analysis of Hic-5 blocked GBR that are dependent on CHD9 or on BRM. De novo motif analysis was performed using HOMER; the top 5 ranked motifs are shown with their p-value, score for concordance of the de novo motif with the identified match, and prevalence near the GBR set examined. (TIF) [file pone.0196965.s003.tif]

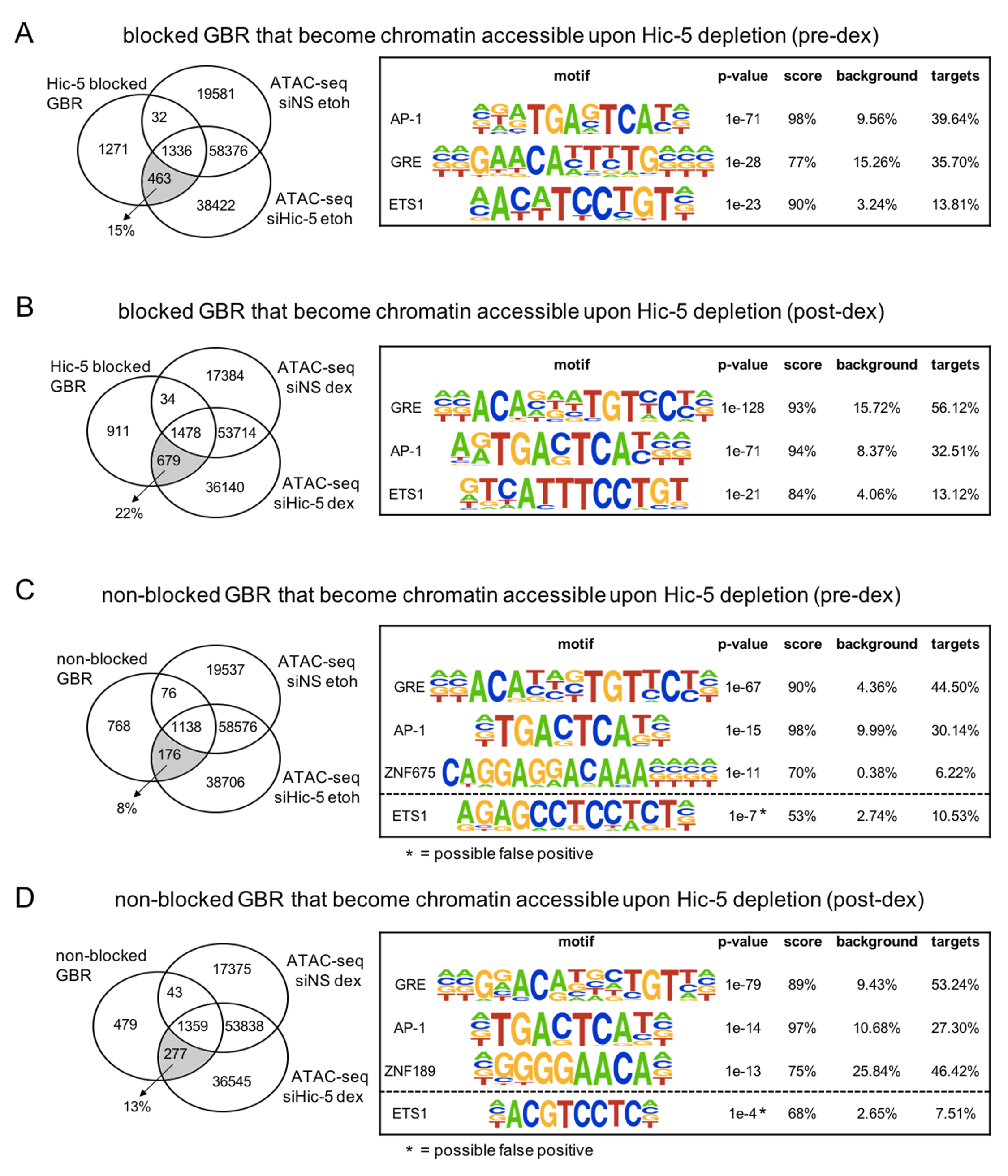

Supplement: S4 Fig — Hic-5 blocked GBR (A-B) or non-blocked GBR (C-D) were overlapped with two other sets: open chromatin regions identified by ATAC-seq in Hic-5 positive cells; and open chromatin regions in Hic-5 depleted cells. This was done for ATAC-seq data from cells treated with vehicle ethanol (etoh, A and C) or cells treated with dex (C and D). The shaded region of each three-way Venn diagram indicates the GBR that become newly chromatin accessible upon Hic-5 depletion. De novo motif analysis was performed on the GBR in each shaded region, using HOMER. The top 3 ranked motifs are shown with their p-value, score for concordance of the de novo motif with the consensus sequence of the identified match, and prevalence near the GBR in the set examined. Motif analysis was performed in a 1-kb window centered on the GBR peak for all GBR belonging to the set examined. Motifs below the dotted lines in C and D (shown for comparison to A and B) are for the highest scoring member of the ETS family, which were not one of the top three motifs and were indicated as possible false positives in C and D by HOMER. (TIF) [file pone.0196965.s004.tif]
